# Dr. Roswell Park’s Anniversary

**Published:** 1909-01

**Authors:** 


**A Monthly Review of Medicine and Surgery.**

*EDITOR:*

WILLIAM WARREN POTTER, M. D.

All communications, whether of a literary or business nature, books for reviewand exchanges, should be addressed to the editor, 238 DELAWARE AVENUE, BUFFALO, N. Y.

VOL. LXIV. JANUARY, 1909. No. 6

### Dr. Roswell Park's Anniversary.

DR. Roswell Park was the guest of honor at a banquet tendered him by the medical profession at the Iroquois Hotel, on the evening of December 7, 1908, in commemoration of his twenty-five years' professorship of surgery at the University of Buffalo.

The committee in charge of the arrangements, Drs. Charles G. Stockton, Charles Cary, M. D. Mann, Eugene A. Smith and H. U. Williams, of which committee Dr. E. R. McGuire, was treasurer, issued invitations to the profession to participate in honoring Dr. Park, and invited some of the prominent surgeons of the United States to be its guests. From other cities came Dr. Frank Billings, and Dr. L. S. McArthur, and Dr. Arthur D. Bevan, of Chicago; Dr. James Bell, of Montreal; Dr. George W. Crile, of Cleveland; Dr. F. S. Dennis, of New York; and Maurice H. Richardson, of Boston.

Covers were laid for 150 in the large banquet hall of the Iroquois Hotel and the dinner was served at 7.30. The arrangements were perfect and no large dinner has been given in Buffalo in recent years, which has compared with this one in point of excellence, simplicity and artistic detail.

The subscribers to the dinner were seated at small tables, at which the general decorative color scheme was carried out.

The dinner cards, exquisitely designed, were most admirably executed specimens of typographic art. The covers were of heavy plate paper of golden brown, the front bearing an engraved vignette portrait of Dr. Park, enclosed in an oval, embossed in silver. The menu and toast list were on thin hand-laid paper of the same tone. The dinner was most carefully selected and was unusual, in that there was an entire absence of those conventional dishes, which find place on the dinner cards of nearly every large banquet. The menu was:

| | |
|---|---|
| Canapé de Caviare | R. P. Cocktail |
| Huitres sur Coquille | |
| Consommé Printanier Royale | Cabinet Sherry |
| Celeri Olives Amandes Salees | St. Estephe |
| Chair de Crabes, à la Newburg | Sauterne |
| Ris-de-Veau scus Cloche Eugénie | |
| Sorbet Bénédictine | R. P. Cigarettes |
| Suprême de Mallard, à l'Orange | Louis Roederer, 1900 |
| Asperge de California, Vinaigrette | |
| Pouding de prunes Glacè | Whiskey |
| Petits Fours | White Rock |
| Fromage Camembert | |
| Café Noir | Cigars |

The cigarettes were silver tipped and bore Dr. Park's monogram in silver with the date 1883 and 1908.

The opposite page carried the toast list, remarkable in its display of careful selection of appropriate quotations.

> This will last out a night in Russia when nights are longest—Measure for measure. These are begot in the ventricles of memory, nourished in the womb of pia mater, and delivered upon the mellowing of occasion.—Love's Labor Lost.

TOASTS.

"O, a little bit of history, a letter and a jug."—Old Song.

Introduction .................................... Dr. Charles Cary

C. "Here's my hand."
P. "And mine with my heart in't."—The Tempest.

Response .......................................Dr. Roswell Park

"You were in presence then and you can witness with me this is true."—Richard II.

Dr. Park in Chicago ...........................Dr. Frank Billings

"And gladly wolde he lerne and gladly teche."—Chaucer

Dr. Park, the Teacher .......................Dr. Eugene A. Smith

"This is an art which does not mend nature. Change it, rather; art itself is nature—so it is."—Winter's Tale.

Dr. Park the Surgeon ....................Dr. Frederick S. Dennis

"Knowing I loved my books, he furnished me from mine own library with volumes that I prize above my dukedom."—The Tempest.

Dr. Park the Author .........................Dr. Matthew D. Mann

"If he be not fellow with the best king, thou shalt find the best king of good fellows."—Henry V.

Dr. Park the Citizen ........................ Mr. Henry R. Howland

"Give me some wine; fill full. I drink to the general joy o' the whole table."—Macbeth.

........................... Dr. Charles G. Stockton.

"These fellows will do well."—2 Henry IV.

The fact that there was no stated toast for Dr. Stockton was commented upon, and yet it was no surprise when the speaker, in responding to the unnamed toast turned to Dr. Park, and in a gracefully worded speech presented him with a magnificent silver ewer of ancient pattern appropriately inscribed. That this presentation was a surprise to the guest of honor, was apparent in his expressions of thanks and appreciation.

Dr. Charles Cary was toastmaster and in presenting Dr. Park, he lightly and almost blithely reviewed the years which have passed since Dr. Park came to Buffalo. He delved into bits of personal history and intimacies in a fanciful way and at the conclusion of his speech, during which he had aroused all the good humor and enthusiasm in the crowded room, he formally presented Dr. Park.

There have been banquets held in Buffalo and elsewhere where there have been more noisy demonstrations; but it is doubtful if there has ever been seen in this city such a demonstration of affectionate regard for a man in private life, as was accorded Dr. Park, when he arose to respond to the greeting of the toastmaster. Everybody in the room was on his feet, napkins were waved in billowing clouds and the clapping of hands was followed by cheers of welcome; not the hysteric cheers or the enthused applause which greets the oratoric hypnotist or the meteoric public figure of fickle fancy. It was none of this; it was a spontaneous outburst of genuine good-will and friendship for a man who by his works and his deeds and his personality, has won the esteem and the love of his fellow workers. Dr. Park's response was one of simplicity, highly characteristic of the man. His words of thanks and appreciation were expressed in simple language and with deep feeling; he reviewed his work in Buffalo and sketched the changes which had taken place in the twenty-five years of his residence here; the marvelous changes, the great advancement which had taken place in surgery and in hospital equipment; he told of the disadvantages under which surgery was performed twenty-five years ago and how, through the generosity of several public-spirited Buffalonians, it had been possible for the Buffalo General Hospital to have one of the finest clinics in this country, and the University of Buffalo to possess a perfectly equipped laboratory for experimental and research work.

Dr. Mann's address dealt with Dr. Park's position in literature and he reviewed in detail the books and the many important monographs which he had contributed to the literature of surgery and medicine.

Dr. Billings's speech was of a more intimate character, for it took his hearers back to the days in Chicago, when modern American surgery was in its earliest stages. He spoke at length of Dr. Park's career in that city, and of the loss which Chicago had sustained when he came to Buffalo to assume the professorship of surgery. There was, at that time, he said, a place for a man in surgery, and it mattered not whether he were in Chicago or Buffalo or any other city; there was one man to occupy that place and that man was Dr. Park.

Dr. Eugene A. Smith's reply to the toast "Dr. Park as a teacher" was gracefully eulogistic. Mr. Howland in speaking of Dr. Park, the citizen, sketched the civic life of the guest of honor and recited the positions he had occupied aside from his professional life, and paid him a well deserved compliment for his interest in matters which bended to the civil and social well-being of the city. Drs. Bevan, Bell and McArthur and Richardson also spoke.

An unexpected addition to the toast list was the introduction of Mr. Gordon Hayd, a member of the senior class in the medical department of the University of Buffalo who, on behalf of his classmates, presented Dr. Park with a silver set in an address, which was excellently phrased and expressive of the affection of the students for their professor of surgery.

During the dinner Dr. James J. Mooney and Dr. Fred C. Busch sang, accompanied by Dr. Prescott LeBreton. One of Dr. Busch's numbers was "A Fragment," composed by Dr. Park.

The dinner was a most graceful tribute to a great man and fittingly commemorated the close of his twenty-five years of labor as a teacher of surgery at the University of Buffalo.

Dr. Dennis in speaking of Dr. Park, the surgeon, paid tribute to the work he had performed and the high position he occupies in both this country and Europe.

---

THE State of New York is making a strong fight against the inroads of tuberculosis. New York State day December 21, at the international exhibition in New York city was a great success. Speakers from various points told of the progress making in their several localities.

The speaker from Buffalo was Dr. John H. Pryor, who summarised the Buffalo situation thus:

Buffalo lacks two paramount requisites which are necessary in a campaign against tuberculosis. There is no provision for institutional care of the early case, and has practically no reporting of those afflicted until dead. Tuberculosis is not attacked as an infectious disease, and thus far the sufferers are not treated as afflicted human beings. We cannot reach, help and control until the consumptive is located, and I do not believe that prevention, unless associated with common sense relief, will ever be successful. Our vaunted knowledge of the cause, mode of care and control becomes manifest largely through a flux of garrulity and an occasional tap with a tack hammer in the dark.

The results are appalling, and the people know it. The usual dose of statistics has been administered. It might be mentioned casually that the saving of life is a well known method of reducing a disgraceful death rate Experience and reflection strengthen the belief that we must find the recent recruit, remove him from the ghastly procession and supply intelligent relief and prevention when it may be effective. We have an association, a day camp, a dispensary and a class system, and we touch a fringe. The vast majority of consumptives die because they are poor.

When we decide to be frank, and tell the whole truth, the people may take a holiday and do something about tuberculosis. They may have to be guided by the medical profession to avoid a panic due to a confusion of ideas, and the multiplicity of beguiling and quaint theories so abundant at present. The size of the problem is no excuse, but the timid no longer belong in the fight. They can trot along behind and yelp about ethics and conservatism. The men to enlist are those who are tired of the frightful ravages of disease and the unnecessary death rattle in the consumptive's throat. They may be ugly enough to tell a community how cruel and inhuman it has been to the consumptive, while it has helped all other dependent sick. I insist that the public has not been taught all the facts.

---

CALOX, the oxygen tooth powder continues to grow in favor with the two professions,—medicine and dentistry. The reason is not difficult of solution. The combination of lime and oxygen in this form assures the most perfect cleanliness of mouth and teeth, destroying pathogenic germs, preventing decay, and rendering dentures germ proof. Calox should be found not only in the family of every physician and dentist, but in every lay family of refinement.

---

THE Moët & Chandon White Seal very dry champagne should be prescribed by physicians wherever such medicine is needed. Its delicate flavor makes it agreeable to sensitive palates and when mixed with Apollinaris, is tolerated by the most irritable stomach. Moreover, it is a wine that should find its way to every table whenever champagne is served.

---

Apollinaris, now as always the queen of table waters, is one of the features of the sick room which should not be overlooked. We esteem it the best carbonated water for the invalid that has yet been offered. Besides being a palatable beverage it furnishes a useful vehicle for many potions or draughts. Apenta is a very useful laxative that should be appealed to whenever such water is needed.

---

